# Supplementary material for: Single Cell Transcriptomic and Chromatin Profiles Suggest Layer Vb Is the Only Layer With Shared Excitatory Cell Types in the Medial and Lateral Entorhinal Cortex
Source: Front Neural Circuits. 2022 Jan 26;15:806154. doi: 10.3389/fncir.2021.806154 (PMC8826650; doi:10.3389/fncir.2021.806154)
Supplement: Supplementary file 14 [file Data_Sheet_1.ZIP › LEC_Sup_vs_MEC_Sup_Motifs/homerResults/motif9.similar.html]

motif9

## Information for motif9

A
G
T
C
C
G
A
T
C
T
G
A
C
G
A
T
A
C
G
T
A
C
G
T
C
T
A
G
A
G
C
T
  
Reverse Opposite:  

C
T
G
A
A
G
T
C
G
T
C
A
C
G
T
A
C
G
T
A
A
G
C
T
C
G
T
A
C
T
A
G
  

|  |  |
| --- | --- |
| p-value: | 1e-30 |
| log p-value: | -6.984e+01 |
| Information Content per bp: | 1.725 |
| Number of Target Sequences with motif | 8539.0 |
| Percentage of Target Sequences with motif | 64.04% |
| Number of Background Sequences with motif | 11788.4 |
| Percentage of Background Sequences with motif | 59.16% |
| Average Position of motif in Targets | 320.5 +/- 233.8bp |
| Average Position of motif in Background | 352.4 +/- 269.2bp |
| Strand Bias (log2 ratio + to - strand density) | 0.1 |
| Multiplicity (# of sites on avg that occur together) | 1.48 |
| Motif File: | file (matrix) reverse opposite |

### Similar de novo motifs found

|  |  |  |  |  |  |  |  |
| --- | --- | --- | --- | --- | --- | --- | --- |
| Rank | Match Score | Redundant Motif | P-value | log P-value | % of Targets | % of Background | Motif file |
| 1 | 0.661 | A C G T A C G T C G T A A C G T A C G T A C G T A C G T T G A C | 1e0 | -1.472599 | 8.53% | 8.35% | motif file (matrix) |
